# Defect Density-Dependent pH Response of Graphene Derivatives: Towards the Development of pH-Sensitive Graphene Oxide Devices

**DOI:** 10.3390/nano12111801

**Published:** 2022-05-25

**Authors:** Shayan Angizi, Xianxuan Huang, Lea Hong, Md Ali Akbar, P. Ravi Selvaganapathy, Peter Kruse

**Affiliations:** 1 Department of Chemistry and Chemical Biology, McMaster University, 1280 Main Street West, Hamilton, ON L8S 4M1, Canada; angizis@mcmaster.ca (S.A.); huangx87@mcmaster.ca (X.H.); hongl1@mcmaster.ca (L.H.); akbarm7@mcmaster.ca (M.A.A.); 2 Department of Mechanical Engineering, McMaster University, 1280 Main Street West, Hamilton, ON L8S 4L7, Canada; selvaga@mcmaster.ca

**Keywords:** pH, graphene, graphene oxide, defect, surface functionalization

## Abstract

In this study, we demonstrate that a highly pH-sensitive substrate could be fabricated by controlling the type and defect density of graphene derivatives. Nanomaterials from single-layer graphene resembling a defect-free structure to few-layer graphene and graphene oxide with high defect density were used to demonstrate the pH-sensing mechanisms of graphene. We show the presence of three competing mechanisms of pH sensitivity, including the availability of functional groups, the electrochemical double layer, and the ion trapping that determines the overall pH response. The graphene surface was selectively functionalized with hydroxyl, amine, and carboxyl groups to understand the role and density of the graphene pH-sensitive functional groups. Later, we establish the development of highly pH-sensitive graphene oxide by controlling its defect density. This research opens a new avenue for integrating micro–nano-sized pH sensors based on graphene derivatives into next-generation sensing platforms.

## 1. Introduction

The pH measurement in an aqueous solution is a universal need and is widely utilized in various applications, from biology [1] to wastewater treatment [2]. The standard techniques for pH detections are often based on electrochemistry (potentiometry) using a porous glass electrode [3,4]. Later, the development of ion-selective membranes introduced a new category of sensing devices, such as field-effect transistors [5,6]. The ion-selective membranes transfer the charge to a conductive active layer under varying gate potentials [7]. Other pH detection methods, including conductometric [8,9] and optical [10,11], operate based on the pH sensitivity of an indicator dye, where adding an organic redox-active indicator is essential. Nevertheless, commercial pH measurements have a number of drawbacks. The porous glass electrodes require high maintenance and are prone to performance loss at low ionic strength or high temperature [12]. The ion-selective membranes are subject to degradation and loss of sensitivity over long-term operation [13,14]. The optical measurements require the introduction of undesirable chemical species in the reaction solution [15,16]. Therefore, developing reagent-free pH-sensitive platforms has remained a relevant topic.

Since the first successful isolation of monolayer graphene from graphite [17], there has been an increasing trend towards integrating these atomically thin structures into many sensing applications, including pH detection units. This broad range of graphene sensing applications is mainly due to three of its characteristics: superior conductivity, high specific surface area, and modulable surface chemistry [18,19,20]. Single-layer graphene (SLG) consists of an sp^2^-hybridized covalently bonded carbon network, while vertically stacked consecutive layers of graphene are held together by van der Waals forces [21]. Since the electronic properties of graphene greatly depend on the presence of delocalized π electrons, any minor disruption of the surface infinite symmetry causes changes in its electrical properties [22,23]. Therefore, defects involving sp^3^ carbon atoms or in-plane lattice asymmetries play a vital role in determining graphene’s ultimate electrical and chemical properties [24]. Accordingly, the defect engineering of graphene can lead to the generation of other graphene derivatives, such as graphene oxide (GO) and reduced graphene oxide (r-GO).

The sensing performance of graphene derivatives in contact with an aqueous environment is profoundly reliant on the electrolyte parameters, including pH, oxidation-reduction potential, ionic strength, dissolved oxygen, and temperature [25]. Therefore, the concept of pH sensors based on graphene derivatives can be fully developed only if the aqueous electrolyte’s impact on graphene is entirely investigated. Moreover, the pH-sensing mechanism of graphene has been shown to be defect-dependent [26], but there is still no clarity on the role of defects in the pH response of graphene devices.

This lack of understanding originates because “ideal” defect-free graphene tends to be hydrophobic [23], making it challenging to elucidate the structure of the aqueous solution/graphene interface [27]. However, oxygen-based impurities enhance the surface local charges and decrease the liquid/surface contact angle [28,29,30]. Therefore, the defectivity level, type of defects, and their configurations (edge or plane) on graphene play a vital role in the graphene–solution interface. Despite a few studies exploring the possibility of developing a pH-sensing platform based on graphene derivatives [28,31,32,33,34,35], there is still a gap in understanding their response to pH at high defect levels. Thus far, there exist only a few reports on the application of GO in pH sensing, primarily due to three main limiting factors in the development of such devices: (i) the water dispersibility of the GO due to its hydrophilic nature and high negative zeta potential [36], (ii) its low surface conductance and semiconductive nature, (iii) the lack of understanding of the pH detection mechanisms of GO [37]. In 2020, potentiometric and chemiresistive measurements of hydrothermally reduced GO revealed potential sensitivities of 66 mV/pH (pH 2–12) and 44 mV/pH (pH 4–7), respectively. Accordingly, the reported working range of the chemiresistor was limited to acidic pH, and the higher pH sensitivities required a three-electrode design [38,39]. Although the presence of oxygen functional groups was shown to be responsible for such detection, no explanation for the limited linear range and mechanism has been proposed. In a different study, a sensitivity of 51 mV/pH was obtained for a pH of 2–10 using potentiometry [40] and FETs [41]. The authors successfully demonstrated the reversibility of the charge transfer on the GO surface upon exposure to pH. While the nature of the response has remained unknown, this study has pushed the boundaries toward mono- and diprotic acid potentiometric titration units based on GO.

Herein, we demonstrate the possibility of developing a pH-sensing platform based on various graphene derivatives, including single-layer graphene (SLG), few-layer graphene (FLG), and GO, focusing on the mechanisms by which each of these structures responds to pH. Furthermore, we examine the defect density-dependent pH response of graphene by varying the -COOH, -OH, and -NH_2_ concentrations. The results fill the existing gap in understanding the GO pH detection mechanism and creates foundations for developing reagent-free GO pH chemiresistive sensors with a high sensitivity and reproducibility. The results of this study open up a new window into the development of graphene derivative-based pH sensing devices.

## 2. Materials and Methods

### 2.1. Single-Layer Graphene Transfer Process

The SLG coated with PMMA was purchased from Graphenea Inc. To fabricate the sensor, the PMMA/SLG/Cu samples were cut into 1 cm × 1 cm squares and then placed into 200 mL 0.1 M Ammonium persulfate (APS, purchased from VWR) to etch off the copper film. After about 30 min, the PMMA/SLG samples were transferred to 500 mL DI water to rinse off the remaining APS and transferred on a glass slide (VWR). The samples were first heated to 100 °C for 30 min and then annealed for 2 h at 600 °C under N_2_ using a three-heat zone tube furnace (Lindberg Thermodyne 21100). The samples were then immersed in acetone overnight (16 h) to remove the PMMA. The annealing step is essential to achieve minimum PMMA residue on graphene.

### 2.2. Synthesis of FLG and Sensor Fabrication

**Synthesis:** The synthesis process of FLG has already been reported elsewhere [42]. Briefly, 40 mg of graphite powder (Alfa Aesar, Haverhill, MA, USA, 99.99%) is mixed with 4.5 mL and 10.5 mL of IPA and DI water, respectively. The mixture is then sonicated for 6 h using an Elmasonic P60H ultrasonic cleaner, 100% power; sweep mode at 37 kHz. Then, the products are centrifuged (Eppendorf MiniSpin Plus microcentrifuge) for 5 min at 14,000 rpm (13,149× *g*). Afterward, the supernatant products are collected and centrifuged again for 15 min at the same speed. Lastly, the precipitated products are ordered and used for sensor fabrication.

**Figure 1:**
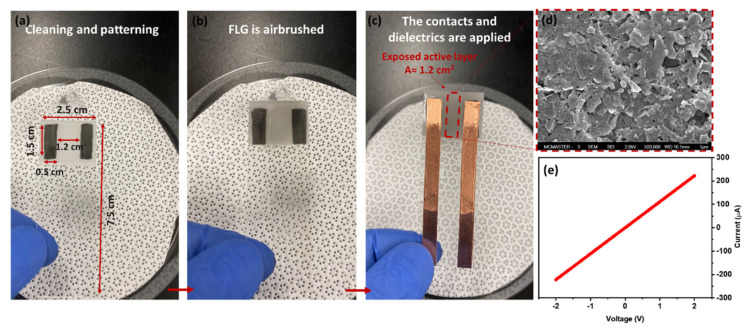
Sensor fabrication steps: (**a**) Cleaning and prepatterning with pencil-drawn contacts, (**b**) airbrushing FLG, and (**c**) attachment of Cu tape and dielectrics. (**d**) SEM image of the FLG airbrushed on the surface, (**e**) the I-V curve of the fabricated chemiresistive sensor indicating the ohmic device (sensor resistance was ~10 kΩ ).

**Sensor fabrication:** The frosted sides of the glass slides (VWR VistaVision) were initially rinsed with methanol (Fisher Scientific Canada, HPLC) and patterned by two parallel rectangles drawn by a 9B pencil (Figure 1a). Then, the FLG suspension was airbrushed (e NEO-Iwata CN Gravity Feed Dual Action Brush #N4500) using a nitrogen gas directly on the surface, preheated to 150 °C, until the resistance was measured in the range of 5–10 kΩ (Figure 1b). The copper tape was attached as sensor contacts to the pencil-drawn rectangle/airbrushed sample. To avoid direct exposure of copper tape to aqueous solutions, the contacts were covered by parafilm (Parafilm “M”, VWR). Increasing the temperature to 70 °C (above the melting point of parafilm) leads to a more uniform coverage (Figure 1c). An SEM image of an FLG film deposited for a sensor and the I-V curve characteristics of a typical device are shown in Figure 1d,e, respectively.

**Annealing:** The airbrushed samples were placed into a furnace under the flow of N_2_/H_2_ (95%/5%) and heated to 500 °C and 350 °C for FLG and GO, respectively. A temperature of 350 °C has been shown as a safe temperature to anneal GO without considerable thermal decompositions or mass loss [43]. The samples are left to cool down gradually overnight in an N_2_ atmosphere.

**Pyrene derivatives functionalization:** Pyrene carboxylic acid (Py-COOH), 1-amino Pyrene (Py-NH_2_), and 1-hydroxypyrene (Py-OH) were purchased from Sigma Aldrich and used without further purification. To dope the samples, the fabricated sensors were placed into solutions of the respective pyrene in acetonitrile overnight (~16 h). At the end, the functionalized sensors were rinsed with pure acetonitrile to remove any excess pyrene.

**pH measurement experiment:** In preparation for the experiments, the devices were initially placed into a 3.42 mM NaCl solution and left overnight to equilibrate with the environment. An initial ionic conductivity above 0.33 mS/cm (equivalent to 3.42 mM NaCl) minimizes possible interferences due to changes in ionic strength when adjusting pH using NaOH (99%-Caledon Laboratories Ltd., Ahmedabad, india, ACS reagent) or HCl (37.2%-Caledon Laboratories Ltd. ACS reagent). The pH of the solution was adjusted by drop-wise addition of NaOH (0.1 M) or HCl (0.1 M) into the system until the desired pH (pH 3 to 9) was achieved. The devices were kept at each pH for 30 min while the current was recorded at intervals of 1 data point every two seconds. The last 60 data points (two minutes) from each step were used for further analysis.

### 2.3. GO Preparation and SENSOR fabrication

The GO powder was purchased from Zentek Ltd (Canada). Initially, 40 mg of GO was dispersed in 15 mL of ultrapure water and sonicated for 1h. The products were airbrushed on a preheated substrate to 200 °C. If annealing was required, the samples were placed at 350 °C for 24 h under N_2_/H_2_ (95%/5%) reducing environment. Then, the copper tapes were attached and subsequently covered by parafilm as dielectric (as discussed in Section 2.2).

### 2.4. Characterization

A Renishaw inVia Raman spectrometer was used to characterize the defect level of the graphene film. A Renishaw 633 nm HeNe laser with 17 mW power output was focused through the 50× objective lens with a spot size of about 1.5 μm. The laser power used for few-layer graphene analysis was 50% to minimize the noise and 5% power for graphene oxide to avoid film damage. The range of the Raman region was from 500 to 3500 cm^−1^ with a spectral resolution of 2 cm^−1^. Spectra were recorded in at least two different spots for each sample to ensure reproducibility.

The XPS analyses were carried out with a Kratos AXIS Supra X-ray photoelectron spectrometer using a monochromatic Al K(alpha) source (15 mA, 15 kV). A charge neutralizer was used on all specimens. Survey scans were collected from an area of 300 × 700 µm^2^ using a pass energy of 160 eV. High-resolution scans used a pass energy of 20 eV. All spectra were charge corrected to the mainline of C 1s (graphitic carbon, 284.5 eV). Spectra were analyzed using CasaXPS software (version 2.3.14).

## 3. Results and Discussions

### 3.1. pH Response of Bare Graphene

**Figure 2:**
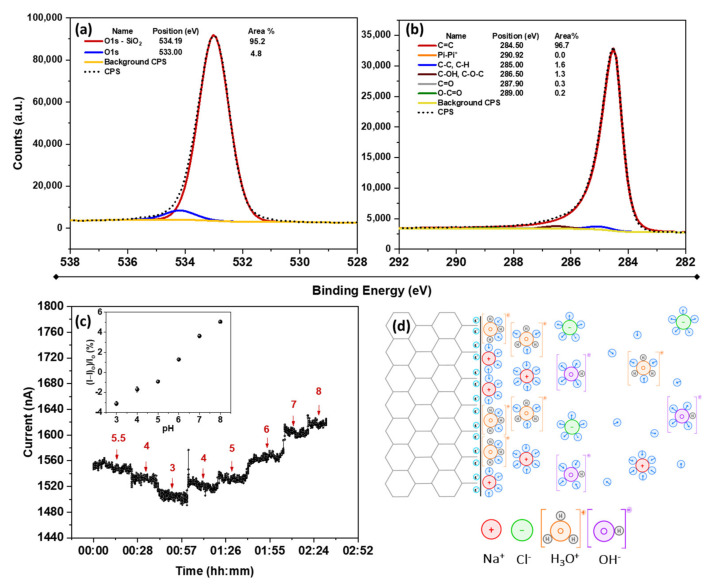
XPS high-resolution spectra of (**a**) O 1s and (**b**) C 1s of SLG, (**c**) pH response of SLG between pH 5.5–8 (the inset shows the corresponding calibration curve) with I_o_ = 1524 nA, and (**d**) schematics illustration of the formation of EDL on graphene in acidic solution and its electrostatic gating charging.

The response of bare graphene to pH was chosen as a starting point for understanding the impact of surface defectivity. Considering that most liquid-phase exfoliation methods to produce graphene derivatives, including ultrasonication, generate a high degree of defectivity (oxygen content between 10–15%), a defect-free (1–2%) CVD-grown SLG was used as a reference sample to obtain an insight into the response of pure graphene to pH. The SLG’s low defectivity was confirmed by the XPS results, as shown in Figure 2. According to the high-resolution O 1s spectra, shown in Figure 2a, the peak at 533 eV is associated with the oxygen doubly bound to C, while the peak at 534.19 eV corresponds to oxygen in the SiO_2_ substrate. The nature of oxygen–carbon bonds can be further analyzed using C 1s high-resolution spectra, as shown in Figure 2b. As seen, C-OH/C-O-C, and C=O peaks located at 286.50 and 287.90 eV are the dominant oxygen-based functional groups. The C=O is often interpreted as a result of graphene’s ketone, aldehyde, and carboxyl groups [44]. The presence of C-OH indicates the formation of a primary or secondary alcohol and carboxyl groups, and the C-O-C implies the appearance of ether and epoxy sites on the surface [45]. These results confirm that oxygen-based functional groups are inevitably formed on graphene during the synthesis or transfer process. In this case, the acetone treatment to eliminate the PMMA and subsequent multiple rounds of bathing in DI water could cause this oxidation. Moreover, the possibility of oxygen-containing contaminants (e.g., carboxylic acids, alcohols, aldehydes, etc.) from the environment cannot be neglected. Nevertheless, the O/C atomic ratio of the transferred SLG is calculated at ~0.05, at the lower end of defect density. The atomic percentage of oxygen and carbon in SLG can be found in the XPS survey spectrum shown in Figure S1a.

The pH response of an SLG chemiresistive device is shown in Figure 2c. The starting pH (5.5) was determined by an equilibrium of the aqueous solution with ambient air, established overnight. Due to the partition of CO_2_ from the ambient air into water, the pH of deionized water gradually equilibrates to 5.6, assuming 400 ppm of CO_2_ in the air. According to Figure 2c, the current through the SLG is observed to decrease when the pH is reduced to 3, and conversely increased when the pH was raised. The low pH response can be explained by the electrostatic gating effect of H_3_O^+^_aq,_ which n-dopes the surface. Since the holes are the majority carrier in graphene due to the presence of electron-withdrawing oxygen atoms, n-doping the surface reduces the charge carriers in the chemiresistor and makes it more resistive. In contrast, electrostatic p-doping of OH^−^_aq_ ions accumulated in the Stern layer increases the current at a high pH [26]. The schematic illustrations of the formation of the electrochemical double layer (EDL) in acidic solutions are shown in Figure 2d. It should be noted that the charge transfers through the formation of the EDL by H_3_O^+^ and OH^−^ are considered fully nonfaradaic. Therefore, the charges are electrostatically gated to the graphene surface. This mechanism is well-defined for SLG and is often deemed the typical graphene response to pH. This phenomenon has been studied in other devices such as FETs [46,47] or Schottky diodes [25], demonstrating the decrease (increase) in Fermi energy upon exposure to a high (low) pH. However, the Fermi energy of SLG is prone to cross the Dirac point upon severe electrostatic doping [48,49]; therefore, it may not be a reliable system to further investigate the role of EDL and defects. Accordingly, the following parts of this research deploy FLG-based chemiresistive devices to explore the role of defects in pH sensitivity.

**Figure 3:**
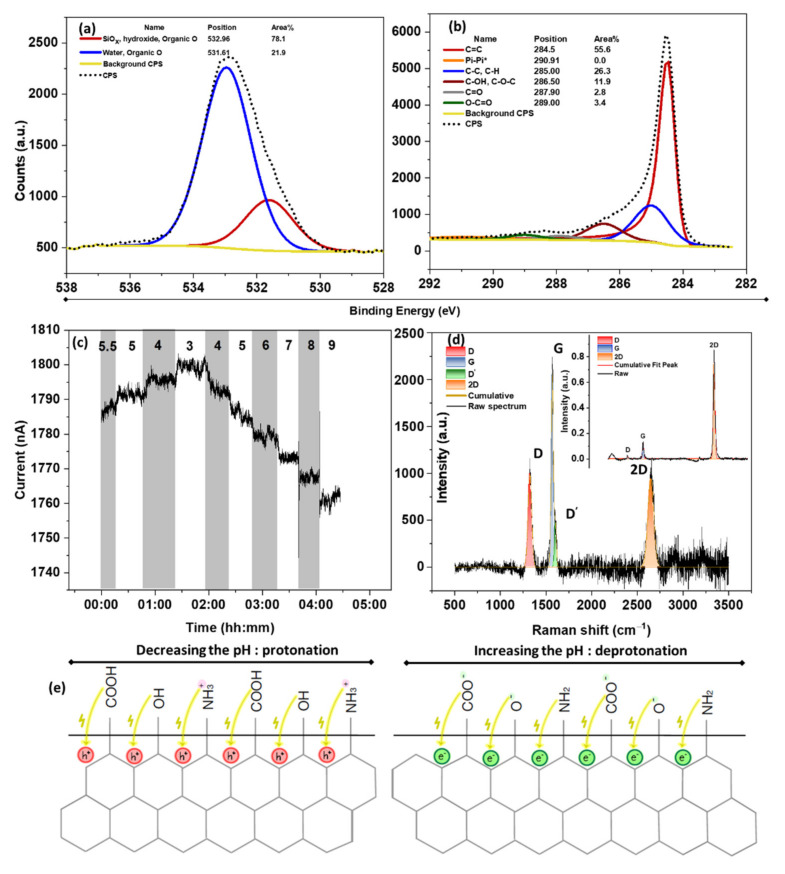
XPS high-resolution spectra of (**a**) O 1s and (**b**) C 1s of FLG, (**c**) pH response of FLG to pH 5.5–9 (I_o_ = 1788 nA), (**d**) Raman of FLG deconvoluted to the main graphene characteristics of D, G, D’, and 2D (inset shows the deconvoluted Raman spectrum of SLG), and (**e**) schematic illustration of defect-induced pH response of FLG through protonation/deprotonation of carboxyl, hydroxyl, and amine groups.

The transition from SLG to FLG requires a careful surface analysis, considering that a higher number of surface defects and functional groups are formed during the liquid-phase exfoliation method. The XPS survey spectrum of the synthesized FLG (Figure S1b) shows 13.9 at% of O and 79.5 at% of C. The O 1s high-resolution spectra (Figure 3a) show a peak at 532.4 eV attributed to trapped water and organic oxygen groups. The larger area of the peaks at 289.0 eV (O-C=O), 287.0 eV (C=O), and 286.5 eV (C-OH, C-O-C) in the C 1s high-resolution spectrum of the FLG compared to the SLG (Figure 3b) exhibit a higher oxygen content in the FLG lattice. Accordingly, the O/C ratio was calculated to be 0.24 by considering the areas of the C=C (284.5 eV) and O 1s (532.4 eV) features in the high-resolution spectra multiplied by their corresponding atomic percentages derived from the survey spectra. A summary of the oxygen-based functional groups in the SLG and FLG can be found in Table 1, demonstrating the greater defectivity of the FLG compared to SLG.

To explore the impact of the enhanced defectivity on the pH response, the chemiresistive response of the FLG is shown in Figure 3c. As seen, the current response to the variation in pH is entirely reversed compared to the SLG (Figure 2c), indicating that the dominant pH response mechanism is different. This means that decreasing the pH reduces the current, while increasing the pH toward the basic solution increases n-doping. We have recently shown that the defect-induced pH response of graphene originates from the protonation/deprotonation of carboxyl and amine at a low pH and hydroxyl groups at a high pH [26]. For example, in carboxyl groups, upon decreasing the pH to 3 (below the pK_a_ = 3.1), the ${-\mathrm{COO}}^{-}\;$is protonated to -COOH, and the surface becomes p-doped. The same concept can be applied to the protonation of -NH_2_ to -NH_3_^+^ and the protonation of -O^−^ to -OH at a pH around 3.7 and 8.2, respectively. Since this charge is transferred directly to the surface, the protonation causes p-doping, while deprotonation results in n-doping, giving an exact opposite behavior to the EDL-induced response. Apart from the defect-induced response, H_3_O^+^ ions have also been shown to enter the gaps between FLG flakes at a low pH. This proton injection in FLG can be confirmed by the blue shift of the 2D peak in the Raman spectra of FLG after the pH exposure (Figure S2). This result could be explained by the Faradaic charge transfer upon proton injection and p-doping of the graphene by lowering its Fermi energy [32,50]. This phenomenon becomes dominant when a porous structure is present [51] (e.g., porous graphitic electrodes in supercapacitors) [50]. Therefore, it is expected to observe a rise in conductance at low pH where H_3_O^+^ is present, mainly close to the surface. Accordingly, the main 2D peak of the FLG spectra (shown in Figure S2) shifts due to the lattice parameter modification by the stiffening/softening of the phonons–charge carrier interactions [52].

The defectivity level can also be estimated by Raman spectroscopy. The FLG (Figure 3d) and SLG (Figure 3d-inset) both exhibit the three main Raman characteristics of graphene, namely D, G, and 2D bands [53]. The D band stands for the presence of sp^3^-hybridized environments, generated mainly by defects. The G band, however, represents the sp^2^ hybridization of the graphene lattice. Thus, the ratio of intensity (or area) of D to G qualitatively characterizes the defectivity level of the structure. Accordingly, the I_D_/I_G_ of the SLG is calculated at 0.11, while the FLG shows 0.43, supporting the XPS results. These values can also be well-fitted to previously published reports that low defect density graphene (less than a cross-over point of I_D_/I_G_~0.35) demonstrates positive pH sensitivity (Figure 2c, inset), while the higher I_D_/I_G_ ratio results in inverted pH sensitivity. It should be mentioned that the positive and negative sensitivities are defined relative to the variation of the current with the pH. This cross-over is where the graphene will become pH insensitive. The schematic illustration of such a protonation/deprotonation mechanism can be seen in Figure 3e.

### 3.2. Selective Functionalization of Graphene

Knowing that the type and density of the defects in graphene derivatives determine their pH response, the development of a pH-sensitive device can be achieved by selective functionalization. For this purpose, noncovalently attached pyrene derivatives with various pH-sensitive functional groups were employed as a model system to resemble the graphene surface terminated with pH-sensitive groups [26]. The charge transfer upon protonation/deprotonation of the functional groups is directly transduced to the FLG [46] via the interactions of the π-electron system of the pyrene ring with the FLG surface [54]. The maximum concentration of each pyrene derivative was chosen to give more than 90% surface coverage [55]. Moreover, the low pyrene solubility in water aids in the stability of the functionalization. However, to minimize the impact of the pre-existing defects of the FLG on pyrene functionalization, it is necessary to anneal the FLG samples under a reducing environment to eliminate the functionalities. The Raman spectra of the FLG before (Figure 3d) and after (Figure S3) annealing demonstrate the effective surface defect reduction, decreasing the I_D_/I_G_ from 0.43 to 0.2. This surface defect reduction can be further confirmed by the pH response of the annealed samples (Figure S4). It is observed that the pH–current relationship is inverted compared to the bare (unannealed) FLG, rather more like the SLG (see Figure S5 for the calibration curve). This phenomenon demonstrates that the FLG has been annealed to a low defect state where the EDL response is dominant.

**Figure 4:**
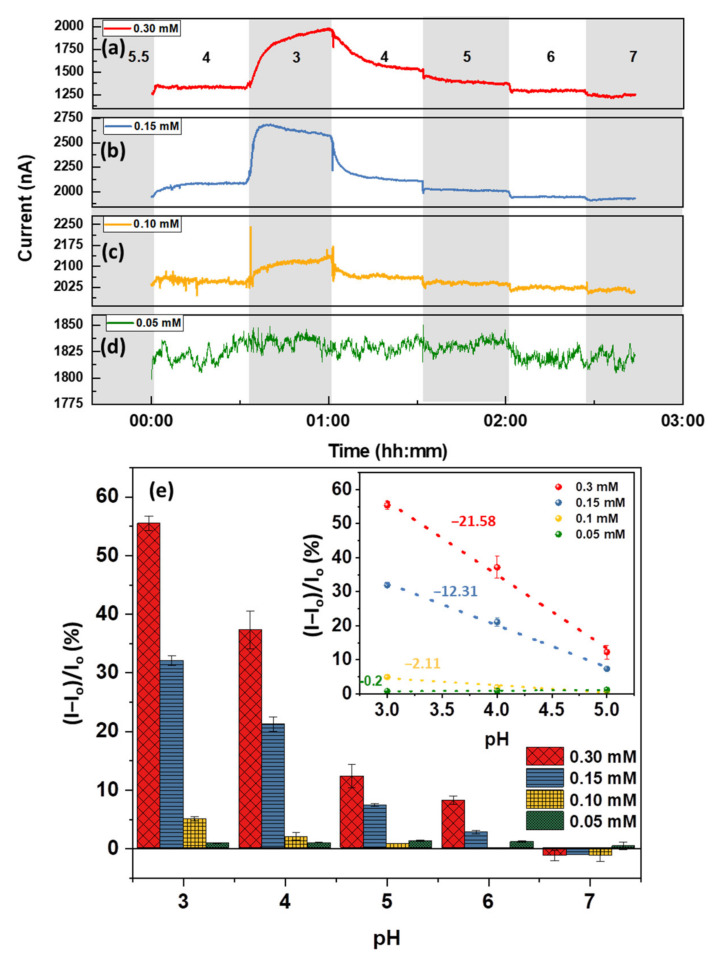
Sensing performance of 8 h annealed FLG functionalized by (**a**) 0.3 mM (I_o_ = 1274 nA), (**b**) 0.15 mM (I_o_ = 1980 nA), (**c**) 0.1 mM (I_o_ = 2052 nA), and (**d**) 0.05 mM (I_o_ = 1812 nA) of Py-COOH; (**e**) the calibration bar graph of the sensors demonstrating the highest pH response at around -COOH pK_a_ (3.1). The error bars represent average ± standard deviation of the last two minutes of the chemiresistive response (3 samples each).

**Figure 5:**
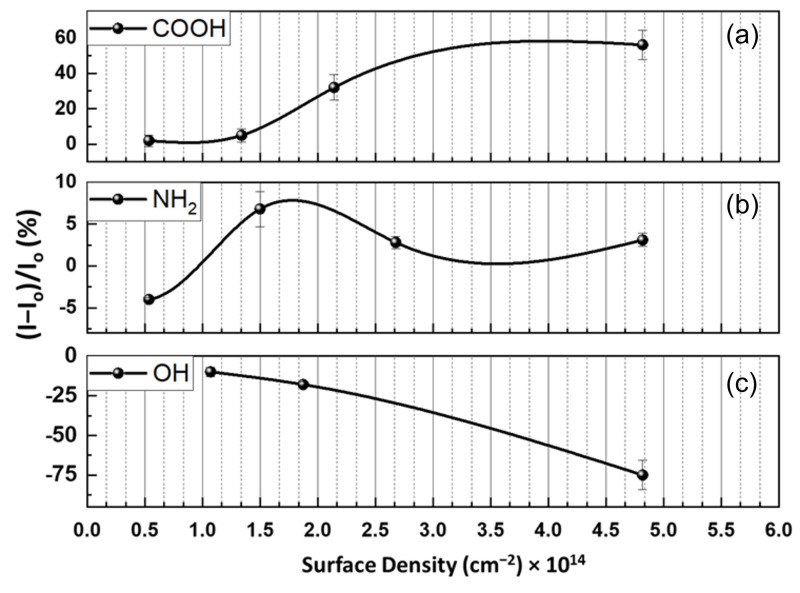
Variation of FLG pH sensitivity as a function of (**a**) carboxyl, (**b**) amine, and (**c**) hydroxyl defect densities. The error bars represent average ± standard deviation of the last two minutes of the chemiresistive response (3 samples each).

Figure 4 displays how the variation of carboxyl group concentration on graphene affects the pH response of a chemiresistive device. The sample exposed to 0.3 M Py-COOH (Figure 4a) demonstrates a selective response to the pH of around 3, giving a current change of ~55% (−21.58%/pH). This response is considered significant compared to the other graphene devices, where the maximum response barely exceeds 20% (see Figure 2c and Figure 3c). The low pH sensitivity of the device at a high pH could be due to two simultaneous factors: (i) the annealing before pyrene functionalization has successfully eliminated the responsive functional groups, or (ii) the pyrene molecules have passivated the existing groups and defects. Notably, the pH sensitivity of the device to a pH range of 3–4 decreases upon reduction of the pyrene concentration to 0.15 mM (−12.31%/pH) and 0.1 mM (−2.11%/pH), as shown in Figure 4b,c, respectively. With doping concentrations around 0.05 mM (Figure 4d), a pH-insensitive platform (−0.2%/pH, Figure 4e, inset) is obtained, indicating the response due to carboxyl groups is balanced out by the response due to electrostatic gating by the EDL. The calibration curves representing the pH sensitivities are shown in Figure 4e. Although it is not easy to accurately measure the defect density of the -COOH group added to the surface, an estimate can be arrived at by making two assumptions: (i) the FLG film is flat with minimum surface roughness; (ii) a monolayer of molecules forms during functionalization. The last assumption has already been validated experimentally and can be considered realistic based on the selected concentrations in Table S1 [55]. Using these assumptions, and considering that each Py-COOH carries one carboxyl group, the approximate surface density of -COOH defects can be estimated:

$$Surface\;density=\frac{SC_{Rel}\;}{A_{Pyrene}}$$

where *SC_Rel_* is the relative surface coverage of pyrene derivatives obtained from the literature [54,55], as shown in Table S1, and *A_Pyrene_* is the area occupied by a single molecule of the respective pyrene derivative. The plot of the maximum pH response of the FLG as a function of the carboxyl group is shown in Figure 5a. Notably, the linear trend is not observed, and a carboxyl group density ~5.36 × 10^13^ cm^−2^ is estimated to be where the EDL becomes dominant. The nonlinear dependence of the maximum response to the carboxyl group surface density confirms that the pH response is not exclusively determined by the functional group mechanism. It should also be noted that any sensing data above the maximum concentration of Py-COOH (0.3 mM) may not be valid due to exposure of the FLG to a concentrated solution that may contain dimers or stacked molecules, leading to an invalidation of the above-noted assumption that only a monolayer of molecules is formed [53,54]. The detailed information of the data shown in Figure 4e and Figure 5 have been provided in Tables S2 and S3, respectively.

**Figure 6:**
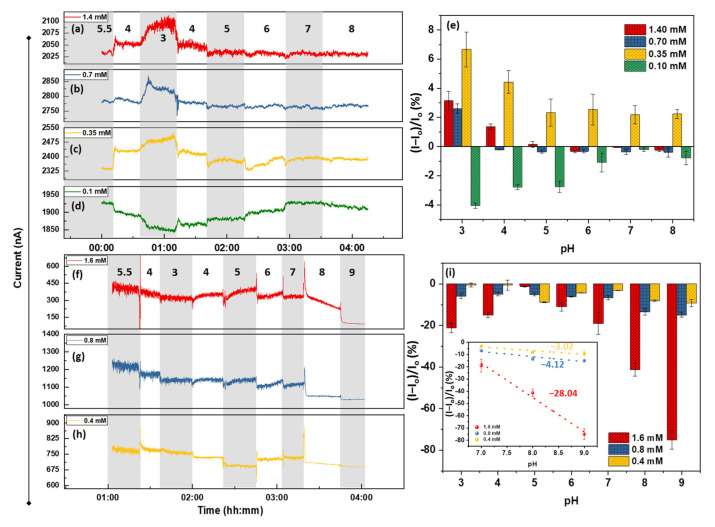
Sensing performance of 8 h annealed FLG functionalized by (**a**) 1.4 mM (I_o_ = 2035 nA), (**b**) 0.7 mM (I_o_ = 2775 nA), (**c**) 0.35 mM (I_o_ = 2330 nA), and (**d**) 0.1 mM (I_o_ = 1927 nA) of Py-NH_2_. (**e**) Graph represents the calibration curve of the Py-NH_2_-functionalized sensors. The sensing performance of 8 h annealed FLG functionalized with (**f**) 1.6 mM (I_o_ = 385 nA), (**g**) 0.8 mM (I_o_ = 1220 nA), and (**h**) 0.4 mM (I_o_ = 764 nA) of Py-OH. (**i**) The calibration bar graph of the sensors demonstrating the maximum pH response at pH around -OH pKa (8.7). The error bars represent average ± standard deviation of the last two minutes of the chemiresistive response (3 samples each).

Similar results can be obtained by using Py-NH_2_, resembling amine groups of the FLG (see Figure 5b). The current increase at a pH of around 3–4 can be interpreted as the protonation of -NH_2_ to -NH_3_^+^ and the p-doping of the surface. Remarkably, the highest concentration of Py-NH_2_ (1.4 mM) does not lead to the maximum pH response of the surface (Figure 6a–d); this occurs at concentrations of around 0.7 mM, giving a maximum pH response of 6.8% (Figure 6e). Moreover, Figure 5b and Figure 6e both reveal two phenomena: (i) 0.1 mM Py-NH_2,_ equivalent to a surface density of ~ 5.35 × 10^13^ cm^−2^ for -NH_2_ groups, is not sufficient to overcome the EDL response of the surface, so that a maximum negative response of ~−4.5% is obtained; (ii) at 0.35 mM (equivalent to 1.5 × 10^14^ cm^−2^), a response of the device to both pH 3 and 4 is considerable, demonstrating that the lower surface coverage of Py-NH_2_ may expose the leftover carboxyl group from the annealing process. It should be noted that the maximum response to pH drops from 3.1% to 2.7%, corresponding to 1.4 mM and 0.7 mM, respectively. Therefore, as expected, lower Py-NH_2_ exposure leads to a lower response to a pH of 3. However, this trend is violated below 0.7 mM, equivalent to the surface density of 2.68 × 10^14^ cm^−2^. One possible mechanism for the lower pH response of the high amine concentration could be an amide formation reaction between -NH_2_ and -COOH of the surface. Upon these reactions, C in R-COOH is reduced to R-CONH_2_, n-doping the surface. This n-doping counteracts the p-doping of the protonation and leads to a lower response.

The pH responses of the Py-OH-functionalized FLG at three different concentrations of 1.6, 0.8, and 0.4 mM are presented in Figure 6. The device pH response is dominated by the protonation/deprotonation of the -O^−^/-OH groups at ahigh pH (pK_a_ = 8.7). Accordingly, the current drop at a pH of 9 of Figure 6f–h is due to this phenomenon. The corresponding calibration curves demonstrate that the device response to a pH of 7–9 is proportional to the -OH concentration and the density, so that a pH of 7–9 and sensitivity of 1.6, 0.8, and 0.4 mM can be calculated for −28.04, −4.12, and −3.02%/pH, respectively (Figure 6i, inset) Furthermore, the sensing behavior of the OH-functionalized FLG can be further analyzed: from a pH of 5.5 down to 3 and back up to 7, the device resembles the annealed FLG, while at a pH of 8 or above, it responds with OH groups. This manifestation reveals that even one sensor can respond differently at different pH ranges, depending on the dominant pH-sensing mechanisms. This result can be further confirmed by the significant impact of the high -OH concentration on the pH response, leading to an almost −75% response to a pH of 9 when the surface defect density is 1.07 × 10^14^ cm^−2^ (Figure 5c). The detailed information of Figure 6e,i can be found in Tables S4 and S5, respectively.

### 3.3. pH Response of GO and Its Application towards the Development of GO-Based pH Sensors

**Figure 7:**
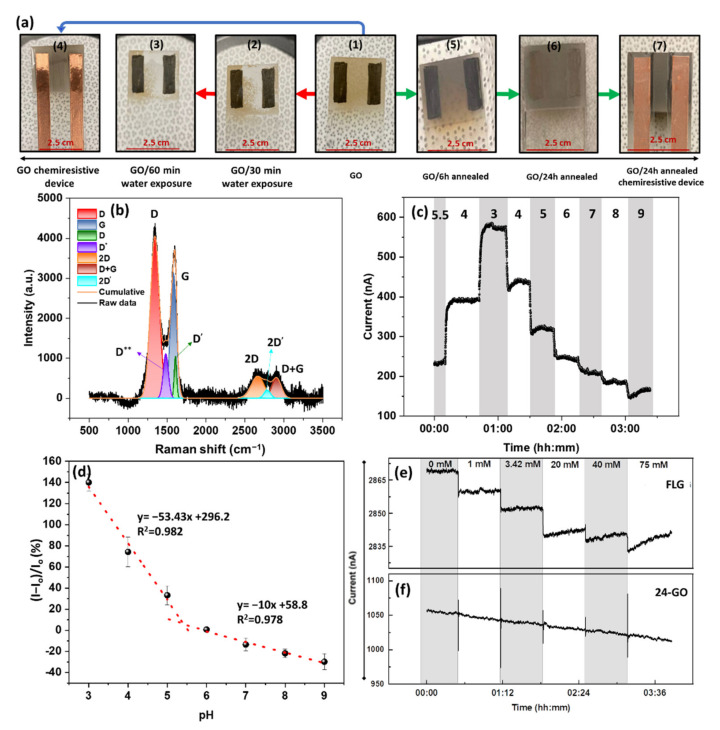
(**a**) The GO sensor fabrication: (**1**) Bare GO; (**2**) Bare GO exposed to the aqueous solution for 30 min; (**3**) Bare GO exposed to the aqueous solution for 6 min; (**4**) tTe GO chemiresistor without water exposure; (**5**) GO annealed for 6 h and (**6**) 26 h; (**7**) The 24 h annealed GO-based chemiresistor. (**b**) Deconvoluted Raman spectrum of 24 h-GO represents the presence of (from left to right): D, D*, G, D’, 2D, 2D’, D + G, and an I_D_/I_G_ ratio of 1.3. (**c**) The pH response and (**d**) calibration curve of 24 h-GO (I_o_ = 242 nA). The solution conductivity response of (**e**) FLG (I_o_ = 2870 nA) and (**f**) 24 h-GO-based devices (I_o_ = 1050 nA).

Based on the discussion above, enhancing graphene defectivity leads to a more defect-induced response and higher sensitivity. Therefore, highly defective graphene derivatives such as GO, carrying various surface/edge functional groups, are expected to be a worthwhile platform to study. The Raman spectrum of the GO with overlapping D and G bands is shown in Figure S6, demonstrating a Raman spectrum consistent with the literature. The Lorentzian deconvolution of the spectrum reveals the presence of multiple subpeaks under the curves, demonstrating an I_D_/I_G_ ratio of 1.74. The significant overlap of the D^’^ and G bands are also due to the enhanced intervalley scattering in the high defect region. Even though the presence of D** at 1479 cm^−1^ is often indicative of disordered carbons (amorphous), it could be due to the cumulative scattering of C=C stretching in sp^2^ regions and the C-H wagging modes in a nanocrystalline diamond [56,57]. The higher defect density can enhance the GO pH sensitivity for aqueous solution applications. However, the stability of GO in water becomes an issue upon its exposure to aqueous solutions. The higher degree of local surface charges caused by functional groups decreases the GO water contact angle, increasing the GO dispersibility. The instability of GO-based chemiresistive device upon exposure to water can be seen in Figure 7a(1–3). Figure 7a(1) shows the airbrushed GO on the glass slide, and (b and c) display the same sensor after exposure to an aqueous solution after 30 and 60 min of exposure, respectively. This means that the delamination of the active layer of GO-based chemiresistors (Figure 7a(3)) limits their stability to less than 30 min. Moreover, the semiconductive nature of GO causes a high film resistance, which is impractical as a conductive active layer. Therefore, despite the remarkable properties of GO, it cannot be used as the active layer in a sensor in its pristine form.

To prevent disintegration, the GO samples were annealed at 350 °C under a reducing environment [43]. Exposure of GO to temperatures above 450 °C should either be done in vacuum systems or under a high flow of inert gas to avoid thermal decomposition [58]. This annealing treatment results in a visible color change from brown (Figure 7a(3)) to gray (Figure 7a(5–7)), indicating the successful reduction of the GO.

A comparison of the I_D_/I_G_ ratio of pristine GO (Figure S6) with that of the 24 h annealed GO (Figure 7b) confirms an increase in surface sp^2^ hybridization. However, due to the surface-insensitive nature of the Raman measurement, the obtained I_D_/I_G_ = 1.3 (Figure 7b) indicates that a considerable number of defects still remain in the bulk. The low-temperature annealing of GO assists in the formation of a stable conductive GO film, while the intrinsic characteristics are preserved. It should be noted that the thermal annealing (reducing) of GO results in a more uniform and well-controlled product as compared to the chemically reduced GO [59]. Moreover, the I_D_/I_G_ = 1.3 obtained from the 24 h-GO reveals that only a surface reduction has occurred, and the term reduced GO is not applied here.

The chemiresistive pH response of the 24 h annealed GO (24 h-GO) is shown in Figure 7c. Notably, the stepwise variation of the current in the 24 h-GO resembles the FLG response; however, the magnitude of the response is much higher. According to the calibration curve shown in Figure 7d, a more than 140% change in the current is obtained by changing the pH from 5.5 to 3, leading to a total response of 175% from a pH from 3 to 9. In order to allow for a calculation of sensitivity despite the overall nonlinear response, the working performance of the device is divided into low (3–5) and high (6–9) pH ranges. The selection of these two regimes is not arbitrary. The low range is chosen based on the pH response of -COOH and -NH_2_, while the latter is based on the pH response of -OH groups. Accordingly, the low range offers a sensitivity of −53.4%/pH. In contrast, the high range sensitivity is calculated as −10%/pH. This difference in sensitivity is due to the highly favorable formation of -COOH during the GO synthesis and the dominant response of the carboxyl groups. Notably, the initial formation of -OH upon the oxidation of graphene to GO could subsequently produce -COOH through the two-step oxidations of hydroxyl → aldehyde → carboxyl [60].

As one of the possible interferences for the pH measurement is the ionic strength of the solution, the performances of the FLG and the 24 h-GO were measured against the changing the solution conductivity. For this purpose, NaCl was used to adjust the solution conductivity, giving up to 7.25 mS/cm for 75 mM. As seen in Figure 7e, the addition of NaCl gives rise to a small stepwise reduction of the current in the FLG. The mechanism justifying this behavior originates from the formation of an EDL on the SLG or FLG due to the long-range arrangement of Na^+^ ions in the Stern layer. Therefore, the electrostatic gating charge screening is the primary mechanism, and the addition of Na^+^_aq_ induces negative charges in the FLG. In contrast, the addition of NaCl does not affect the current through the GO film, and a prolonged drift in current is observed (Figure 7f). The cause of this drift is unclear at this point but could be due to a number of reported effects, including interactions of sodium ions with the oxygen sites over time [61] or variations in the concentrations of dissolved gases (such as CO_2_ or O_2_) as a function of the ionic strength [62]. After drift correction, however, there is no discernible response of the devices to ionic strength. In fact, the EDL response is negligible compared to the high density of defects present in GO-based devices; therefore, they exhibit an inherent potential for the selective detection of pH.

## 4. Conclusions

We have reported the development of a highly pH-sensitive platform based on thermally annealed GO for next-generation sensing devices. To understand the sensing principle of the proposed platform, we established the pH detection mechanisms of the two most commonly used graphene derivatives (i.e., single-layer and few-layer graphene). The contrast in pH responses of the former as a defect-free model (I_D_/I_G_~0.1, O/C ratio = 0.05), with the latter having a degree of defectivity (I_D_/I_G_~0.43, O/C = 0.24), elucidates the importance of defects in pH-sensing graphene. Therefore, the selective functionalization of graphene using various pH-sensitive functional groups demonstrates graphene’s defect density dependence on pH response. An approximate surface density of 4.82 × 10^14^ cm^−2^ of the carboxyl group on graphene leads to a 55% response with an −21.58%/pH sensitivity for a pH of 3–5. The exact same surface density of the -OH groups, however, results in a −75% change in the current, leading to a sensitivity of −28.04%/pH for a pH of 9–7. To develop a GO-based pH-sensitive platform, we demonstrated the importance of the surface reduction treatment at a relatively low temperature (350 °C) to enhance its durability for long-term operation while retaining its high defectivity. As a result, a pH-sensitive device with a maximum current change of 175% (from a pH of 3–9) was reported, giving the sensitivity of −53.43 and −10%/pH pertaining to the pH range of 3–5 and 6–9, respectively. The proposed platform demonstrates minimum interference with ionic conductivity due to the dominance of the defects and offers a reagent-free pH-sensitive substrate for future pH devices.

## Figures and Tables

**Table 1:** Summary of the oxygen-containing groups obtained from XPS spectra of SLG and FLG.

	SLG			FLG		
	Area %	at% *	Ratio to C=C at%	Area %	at% *	Ratio to C=C at%
C=O	0.3	0.1119	0.003074	2.8	2.22	0.05
O-C=O	0.2	0.0746	0.002049	3.4	2.70	0.061
C-OH/O-C-O	1.3	0.4849	0.01332	11.9	9.46	0.214

* Atomic percentage is obtained by Area × total atomic percentage.

## Supplementary Materials

The following supporting information can be downloaded at: https://www.mdpi.com/article/10.3390/nano12111801/s1, Figure S1: XPS survey spectra of Single layer graphene and few layer graphene; Figure S2: Raman spectra of few layer graphene before and after exposure to pH; Figure S3: Raman spectrum of 8h annealed few layer graphene; Figure S4: pH response of 8h annealed few layer graphene; Figure S5: calibration curve of the pH response of 8h annealed few layer graphene; Figure S6: the Lorentzian deconvolution of GO Raman spectrum; Table S1: Summary of information on Pyrene concentrations and their corresponding relative surface coverage obtained from literature; Table S2: average ± Standard deviation of Py-COOH functionalized sensors to pH range 3-8 (3 sensors each); Table S3: average ± standard deviation of maximum response of pyrene derivative functionalized sensors as a functional surface density (3 sensors each); Table S4: average ± standard deviation of Py-NH2 functionalized sensors to pH range 3-8 (3 sensors each); Table S5: average ± standard deviation of Py-OH functionalized sensors to pH range 3-9 (3 sensors each) [63,64,65].
