# Supplementary material for: Defect Density-Dependent pH Response of Graphene Derivatives: Towards the Development of pH-Sensitive Graphene Oxide Devices
Source: Nanomaterials (Basel). 2022 May 25;12(11):1801. doi: 10.3390/nano12111801 (PMC9181870; doi:10.3390/nano12111801)
Supplement: Supplementary file 1 [file nanomaterials-12-01801-s001.zip › nanomaterials-1728707-supplementary.pdf]

Defect density dependent pH response of graphene derivatives: towards the development of pH-sensitive graphene oxide devices

Shayan Angizi[1], Xianxuan Huang[1], Lea Hong[1], Md Ali Akbar [1], P. Ravi Selvaganapathy[2], Peter Kruse [1], *

[1] Department of Chemistry and Chemical Biology, McMaster University, 1280 Main Street West, Hamilton, Ontario L8S 4M1, Canada; angizis@mcmaster.ca

[2] Department of Mechanical Engineering, McMaster University, 1280 Main Street West, Hamilton, Ontario L8S 4L7, Canada.

Correspondence: pkruse@mcmaster.ca.

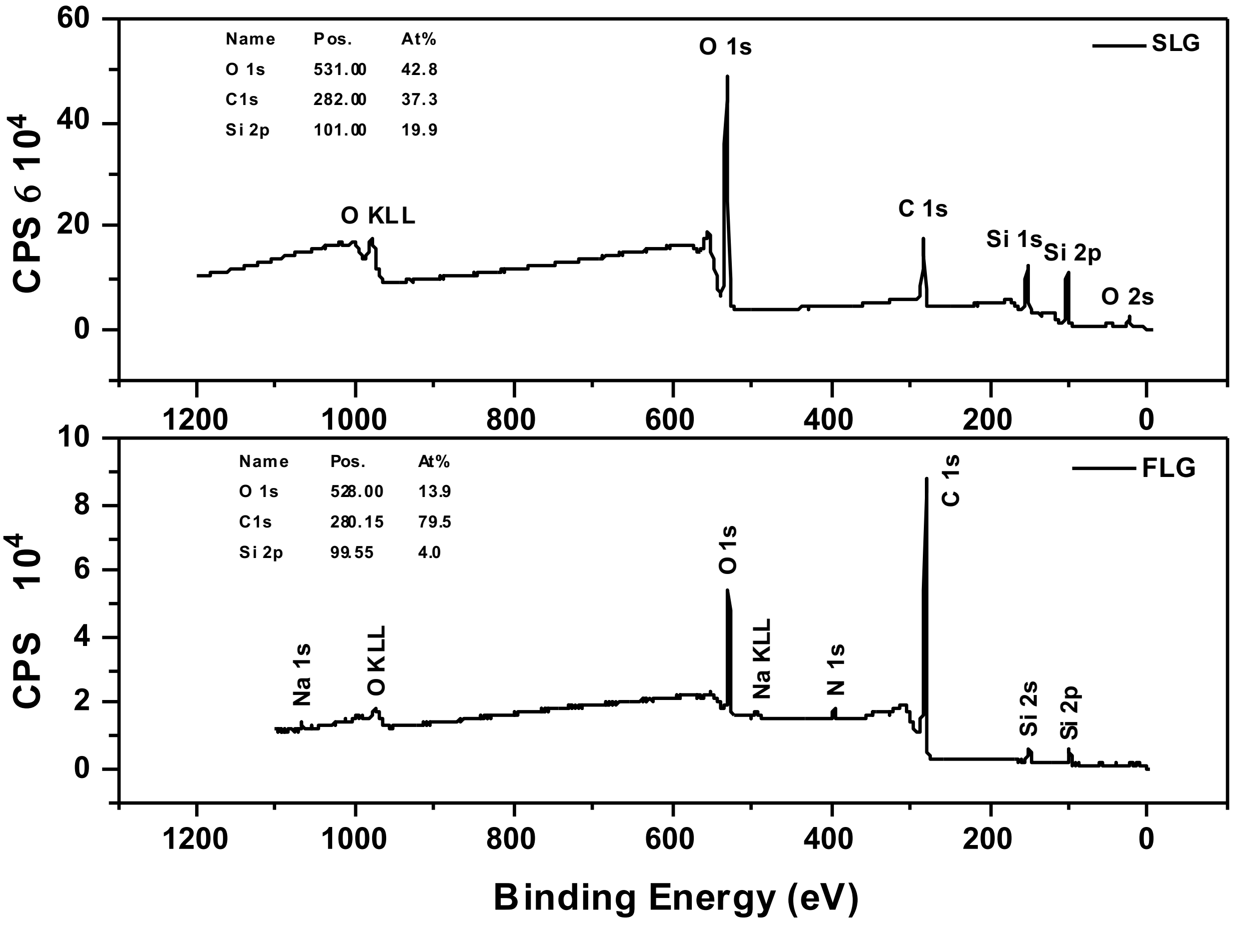

Figure S1. XPS survey spectra of a) SLG, b) FLG

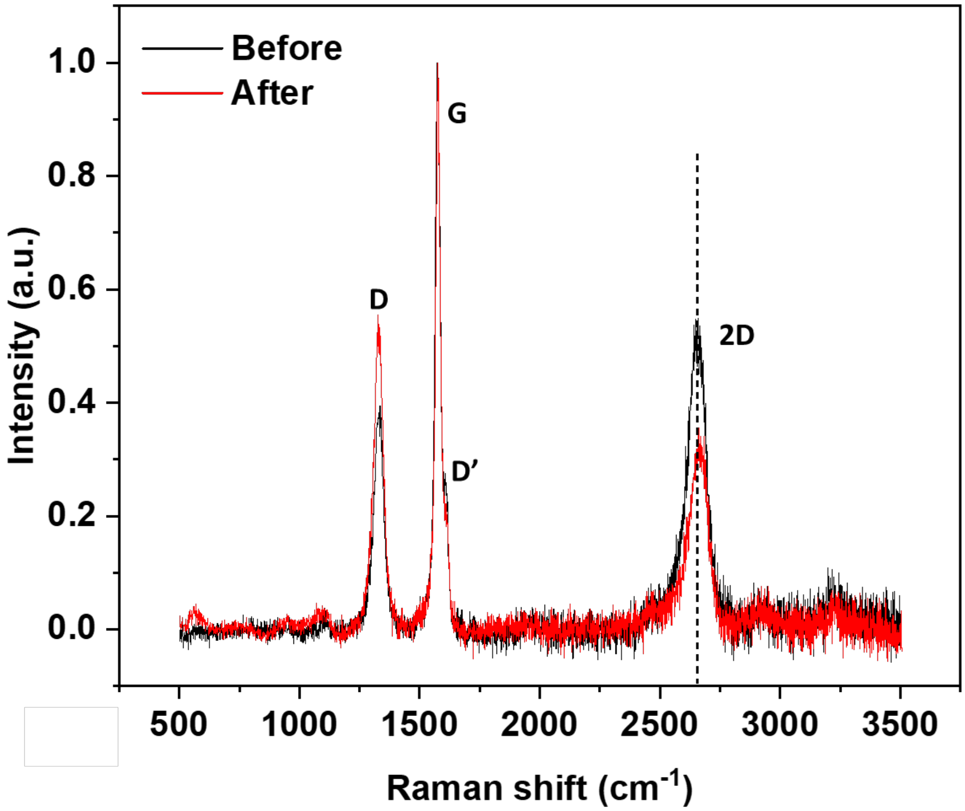

Figure S2. Raman spectra of FLG before and after exposure to pH

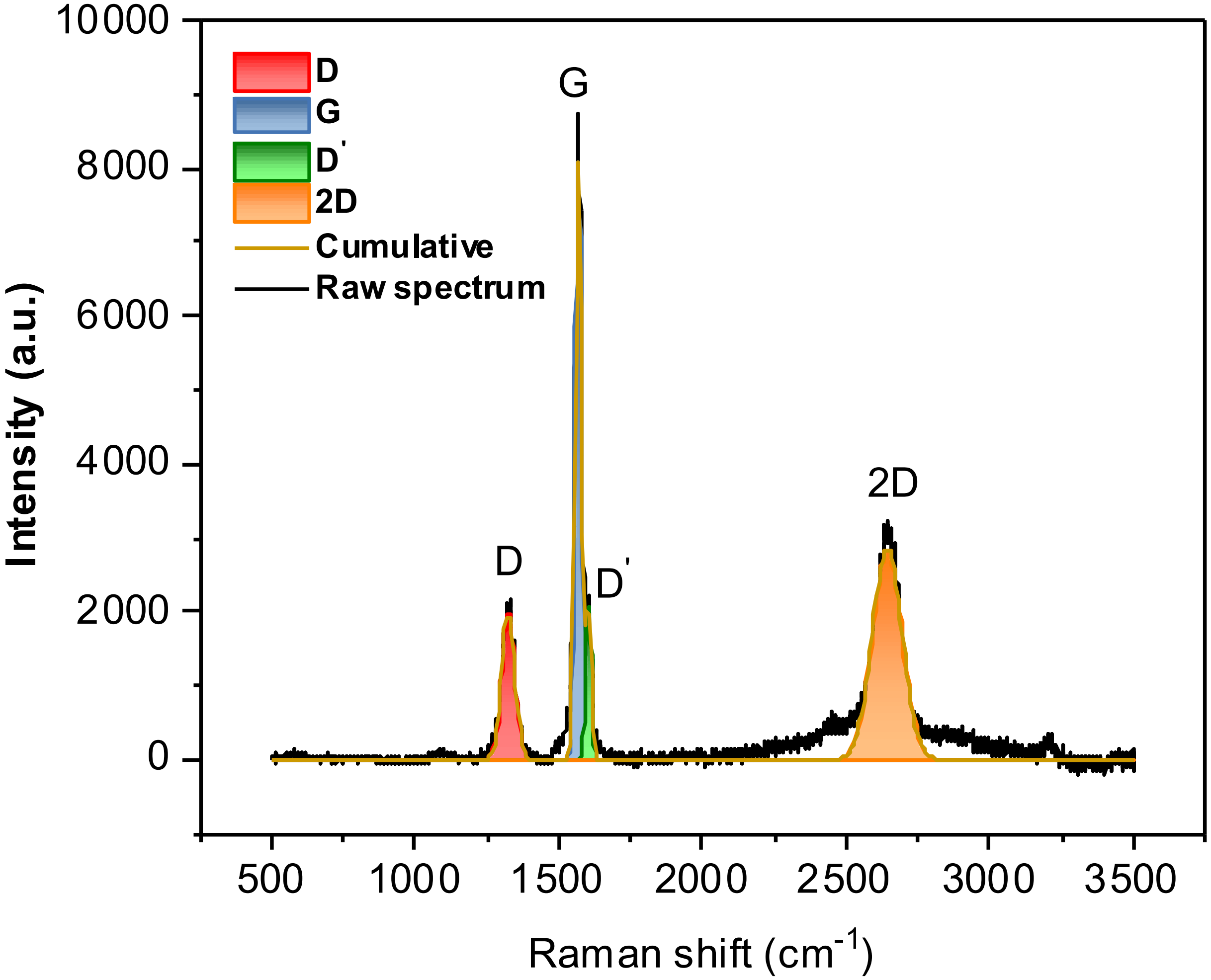

Figure S3. Raman spectrum of 8h annealed FLG

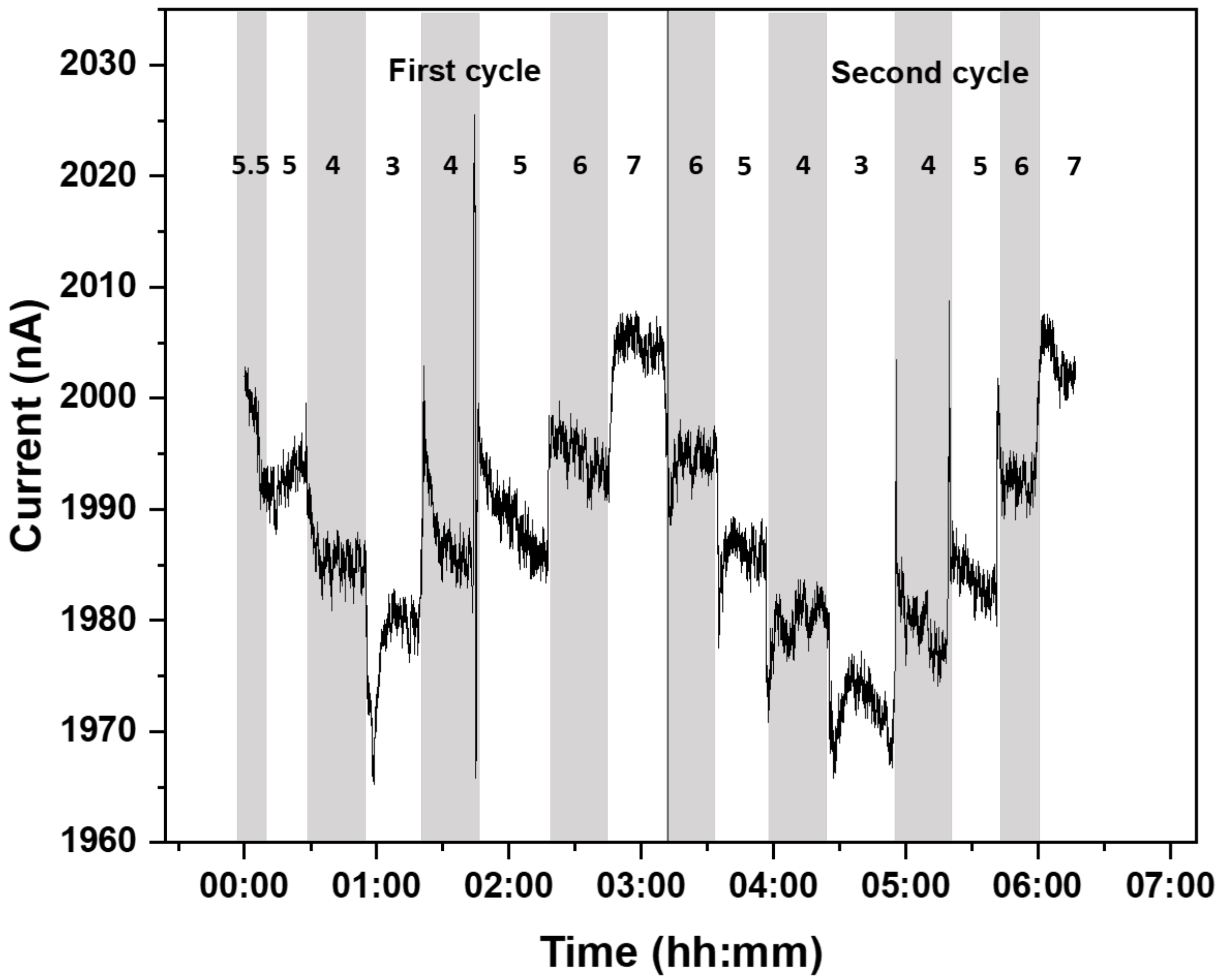

Figure S4. pH response of 8h annealed FLG

Table S1. Summary of information on Pyrene concentrations and their corresponding relative surface coverage obtained from literature [1–3]

	Pyrene derivatives concentration (mM)	Relative Surface coverage (%)	Surface density (#/cm^2)
Py-COOH	0.3	90	4.82×10^{14}
	0.15	40	2.14×10^{14}
	0.1	25	1.34×10^{14}
	0.05	10	5.35×10^{13}
Py-NH_2	1.4	90	4.82×10^{14}
	0.7	50	2.68×10^{14}
	0.35	28	1.05×10^{14}
	0.1	10	5.35×10^{13}
Py-OH	1.6	90	4.82×10^{14}
	0.8	25	1.87×10^{14}
	0.4	20	1.07×10^{14}

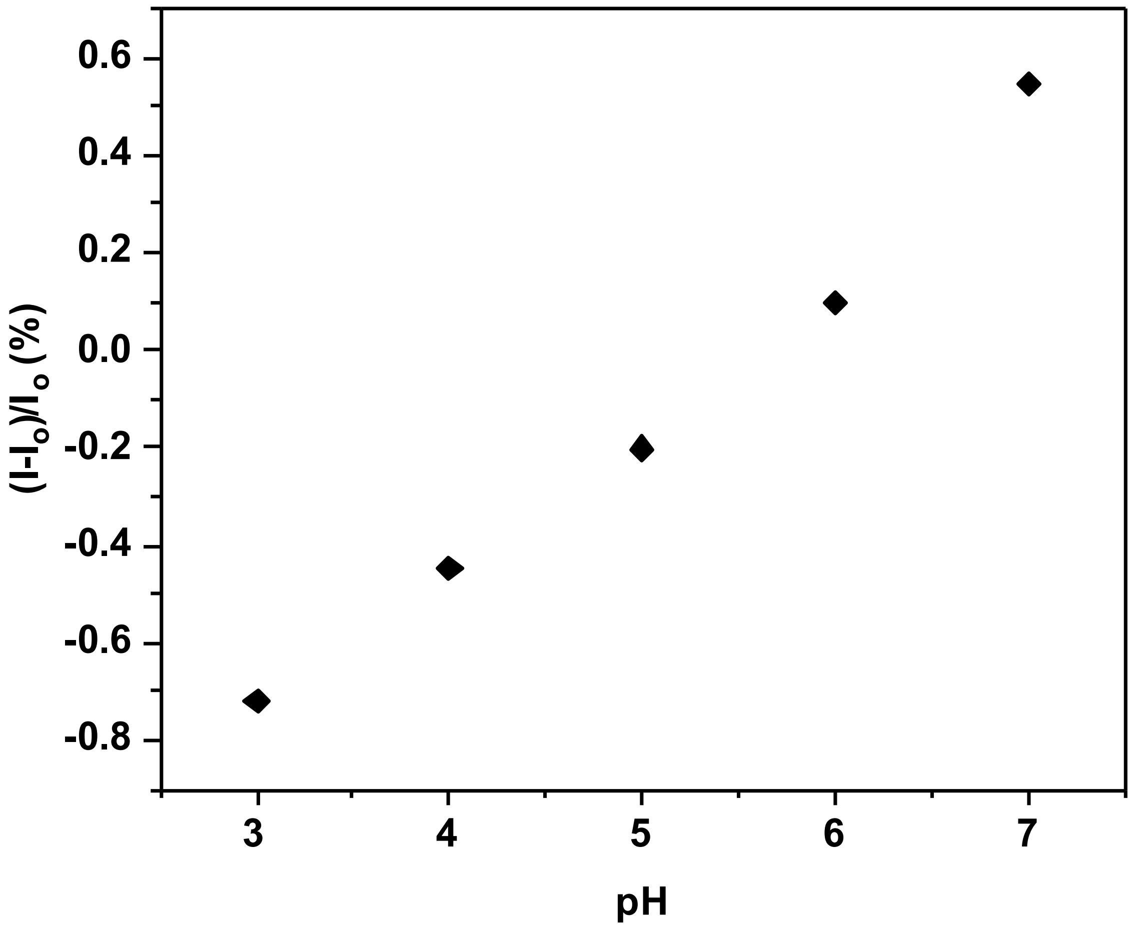

Figure S5. Calibration curve of the pH response of 8h annealed FLG

Table S2. Average ± Standard deviation of Py-COOH functionalized sensors to pH range 3-8 (3 sensors each)

Concentration/pH	3	4	5	6	7
0.30 mM	55.6 ± 1.21	37.2 ± 3.2	12.27± 2	8.09 ± 0.7	-1.33± 0.9
0.15 mM	31.96 ± 0.84	21.12 ± 1.26	7.33 ± 0.24	2.68 ± 0.3	-1.16 ± 0.01
0.10 mM	4.92 ± 0.4	1.9 ± 0.62	0.69 ± 0.02	0.02 ±0.003	1.3 ± 1.1
0.05 mM	0.75 ±0.089	0.84 ±0.044	1.16 ± 0.1	1.04 ± 0.1	0.28 ±0.64

Table S3. Average ± Standard deviation of Maximum response of pyrene derivative functionalized sensors as a functional surface density (3 sensors each).

-COOH		$-NH_2$		-OH	
Surface density	Max. response	Surface density	Max. response	Surface density	Max. response
4.82E+14	56.1 ± 8.31	4.82E+14	3.1 ± 0.63	4.82E+14	-75.1 ± 4.4
2.14E+14	32.4 ± 7.21	2.68E+14	2.6 ± 0.32	1.87E+14	-16.2 ± 1.3
1.34E+14	5.1 ± 3.79	1.50E+14	6.66 ± 1.2	1.07E+14	-9.21 ± 1.8
5.35E+13	2.05 ± 3.26	5.35E+13	-4 ± 0.19	-----	------

Table S4. Average ± Standard deviation of Py-NH_2 functionalized sensors to pH range 3-8 (3 sensors each).

Concentration/pH	3	4	5	6	7	8
1.4 mM	3.1 ± 0.63	1.35 ± 0.21	0.14 ± 0.2	0.34 ± 0.08	0.04 ± 0.008	0.24 ± 0.1
0.7 mM	2.6 ± 0.32	0.21 ± 0.023	0.36 ± 0.091	0.32 ± 0.1	0.36 ± 0.2	0.39 ± 0.32
0.35 mM	6.66 ± 1.2	4.42 ± 0.78	2.32 ± 0.93	2.53 ± 1.05	2.19 ± 0.63	2.23 ± 0.32
0.1 mM	-4.05 ±0.17	2.77 ± 0.168	2.75 ± 0.39	1.09 ± 0.65	0.20 ± 0.126	0.78 ± 0.46

Table S5. Average ± Standard deviation of Py-OH functionalized sensors to pH range 3-9 (3 sensors each).

Concentration /pH	3	4	5	6	7	8	9
1.6 mM	-21.21 ± 2.3	-15 ± 1.2	-1.21 ± 0.2	-11 ± 2.1	-19 ± 5.2	-41.3 ± 3.1	-75.1 ± 4.4
0.8 mM	-5.9 ± 1.2	-5 ± 0.8	-5.2 ± 0.61	-6.11 ± 0.22	6.68 ± 0.9	-13.3 ± 1.6	-14.9 ± 1.3
0.4 mM	-0.52 ± 1	-0.53 ± 2.4	-8.81 ± 0.25	-4.34 ± 0.11	-3.15 ± 0.08	-8.02 ± 0.42	-9.21 ± 1.8

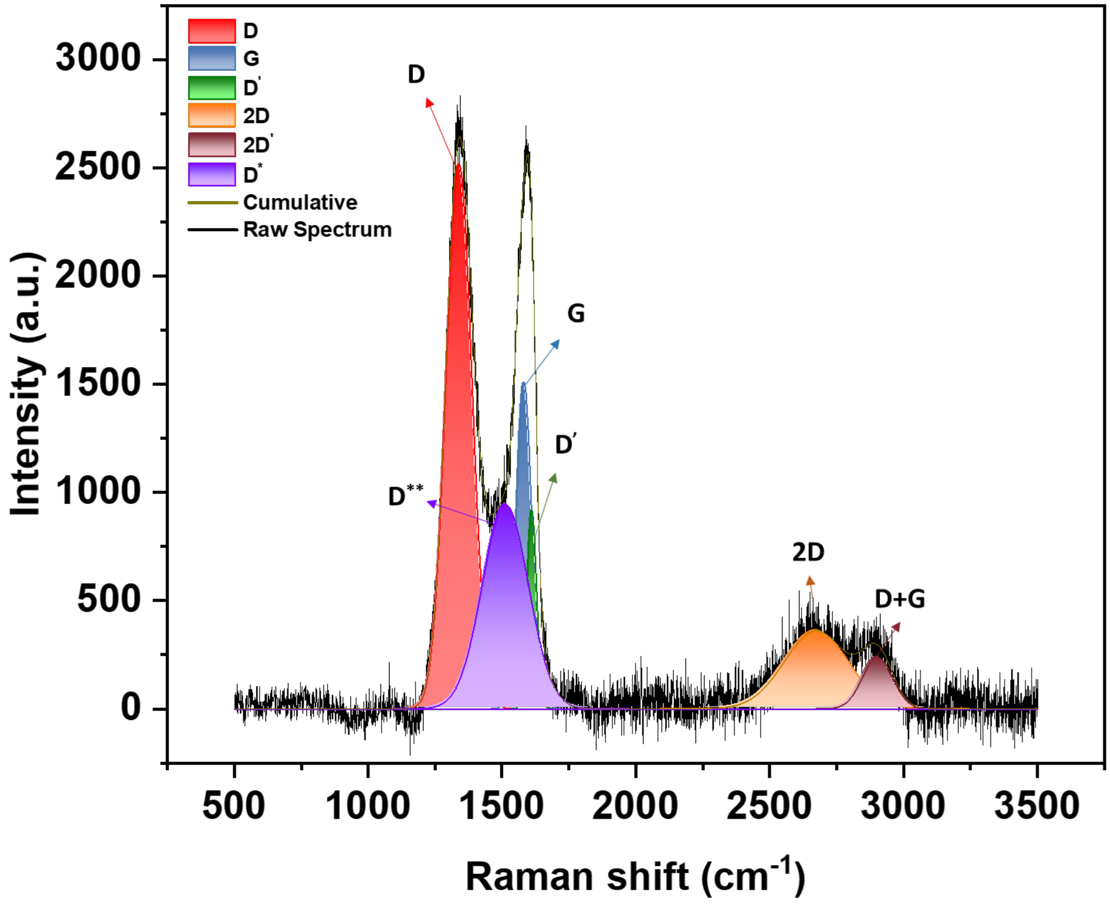

Figure S6. The Lorentzian deconvolution of GO Raman spectrum

References

1. Zhen, X. v.; Swanson, E.G.; Nelson, J.T.; Zhang, Y.; Su, Q.; Koester, S.J.; Bühlmann, P. Noncovalent Monolayer Modification of Graphene Using Pyrene and Cyclodextrin Receptors for Chemical Sensing. *ACS Applied Nano Materials* **2018**, *1*, 2718–2726, doi:10.1021/acsanm.8b00420.

2. An, X.; Simmons, T.; Shah, R.; Wolfe, C.; Lewis, K.M.; Washington, M.; Nayak, S.K.; Talapatra, S.; Kar, S. Stable Aqueous Dispersions of Noncovalently Functionalized Graphene from Graphite and Their Multifunctional High-Performance Applications. *Nano Letters* **2010**, *10*, 4295–4301, doi:10.1021/nl903557p.

3. Georgakilas, V.; Otyepka, M.; Bourlinos, A.B.; Chandra, V.; Kim, N.; Kemp, K.C.; Hobza, P.; Zboril, R.; Kim, K.S. Functionalization of Graphene: Covalent and Non-Covalent Approaches, Derivatives and Applications. *Chemical Reviews* **2012**, *112*, 6156–6214, doi:10.1021/cr3000412.
